# A true response of the brain network during electroacupuncture stimulation at scalp acupoints: An fMRI with simultaneous EAS study

**DOI:** 10.1002/brb3.2829

**Published:** 2022-11-25

**Authors:** Hui Chen, Kay Jann, Yijing Li, Jiling Huang, Yilei Chen, Yingjie Kang, Zhigang Gong, Yanwen Huang, Hui Wang, Songhua Zhan, Wenli Tan

**Affiliations:** ^1^ Department of Radiology Shuguang Hospital Affiliated to Shanghai University of Traditional Chinese Medicine Shanghai P. R. China; ^2^ Laboratory of FMRI Technology (LOFT) Mark & Mary Stevens Neuroimaging and Informatics Institute Keck School of Medicine University of Southern California Los Angeles California USA; ^3^ Department of Acupuncture Shuguang Hospital Affiliated to Shanghai University of Traditional Chinese Medicine Shanghai P. R. China

**Keywords:** brain networks, functional magnetic resonance imaging, scalp acupoints, simultaneous electroacupuncture stimulation

## Abstract

**Objectives:**

The aim of this study was to explore simultaneous brain network responses to electroacupuncture stimulation (EAS) at scalp acupoints by accounting for placebo effects.

**Materials and Methods:**

Sixty healthy subjects were recruited and randomly divided into two groups: Group 1 and Group 2. Functional magnetic resonance imaging (fMRI) was performed in Group 1 with sham acupuncture stimulation at acupoints Shenting (GV24) and Touwei (ST8) without EAS. Group 2 underwent verum EAS at the same acupoints during fMRI. Independent component analysis was used to analyze the fMRI data. Full‐factor statistical analysis was used to compare the differences in fMRI data between the two groups and evaluate the changes in functional connectivity in brain networks after verum electrical stimulation (Group 1 [after sham electrical current stimulation − before sham electrical current stimulation] − Group 2 [after verum electrical current stimulation − before verum electrical current stimulation]) (*p* <.001, extent threshold *k* = 20 voxels).

**Results:**

Six brain networks were identified. Significant increased functional connectivity was observed in the right and left executive control networks, sensorimotor network, and attention network, while decreased functional connectivity was mainly found in the default mode network. There were no statistically significant differences in the salience network.

**Conclusions:**

fMRI with simultaneous EAS provides a method to explore brain network responses due to EAS at scalp acupoints. The networks responsible for cognition are differentially activated by EAS in a coordinated manner.

## Graphical abstract

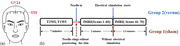

## 1 INTRODUCTION

Acupuncture is an important component of traditional Chinese medicine (TCM) and has been accepted as an effective therapy for many diseases, especially neurological diseases (Thirthalli et al., 2016; Zhao et al., 2017, 2019). Because the stimulation parameters are easily controlled and the stimulation models are correspondingly stable, the clinical application of electroacupuncture stimulation (EAS) has become popular (Napadow et al., 2005; Zhang et al., 2014a). A number of studies have confirmed that EAS produces better subjective evaluations of treatment effects than traditional acupuncture (Lin et al., 2018; Schliessbach et al., 2011).

Recently, functional magnetic resonance imaging (fMRI) has been widely utilized to explain changes in brain connectivity after EAS (Mawla et al., 2021). Studies in healthy volunteer subjects suggest that EAS can regulate connectivity in somatosensory, cognitive, affective, and memory‐related regions (Shi et al., 2017). The analysis of brain networks based on regions of interest (ROIs) could significantly enhance our understanding of the effects in the brain induced by EAS. To date, most scientists working in the field of fMRI for acupoint research have mainly focused on resting‐state fMRI (rs‐fMRI), which involves scanning before or after acupuncture (Cai et al., 2018; Shi et al., 2021a; Zheng et al., 2018). However, brain function is vulnerable to the influence of the external environment, noise, light, and psychological status, and there are some pseudo activations in the brain cortex across two rs‐fMRI scans. Therefore, real‐time scanning of brain areas during EAS is the best means to exclude interference by these factors. More recently, current acupuncture theory has suggested that changes in functional connectivity (FC) during acupuncture are dynamic (Qi et al., 2020). fMRI with simultaneous EAS may add relevant information to understand the effects of EAS as it more accurately represents the dynamic nature of the brain connectivity produced by EAS. This fMRI, similar to task fMRI, was designed to impose specific EAS stimulation to observe the corresponding response in certain parts of the cerebral cortex (Duif et al., 2020; Sakai & Passingham, 2003), by which real‐time changes of brain regions' connectivity during acupuncture can be obtained. Therefore, fMRI with a simultaneous EAS approach may help clarify the neurobiological substrates induced by EAS. Currently, the application of fMRI with simultaneous EAS for acupoint research has mainly focused on acupoints in the upper and lower limbs because these acupoints are easier and more feasible to manipulate during MR scanning than scalp acupoints, such as Hegu (LI4), Zusanli (ST36), and Yanglingquan (GB34) (Chen & Liu, 2019; Cho et al., 2013; Napadow et al., 2013). However, researchers have found that acupuncture at scalp acupoints is more likely to change brain function than acupuncture at upper and lower limb acupoints (Park et al., 2009), and acupuncture at scalp acupoints has advantages over body acupuncture in clinical and research settings (Yau & Ip, 2019). Based on principles of acupoint selection with respect to neighboring brain regions, there are extensive clinical applications of scalp acupuncture to treat various central nervous system diseases (Chung et al., 2019; Jiang et al., 2018; Lee et al., 2013). Therefore, online stimulation of EAS during fMRI is meaningful for clarifying the immediate effect of scalp acupuncture in brain networks.

In clinical practice, acupuncture treatment usually involves a combination of at least two acupuncture points. Thus, investigation into the relationships between needling and the cerebral network should involve a combination of acupuncture points (Zhang et al., 2013). Acupoints Shenting (GV24) and Touwei (ST8) were chosen for the intervention in this study for two reasons. First, GV24 and ST8 acupoints are in the projection area of the frontal and temporal lobes, which are closely related to advanced thinking, spirit, and memory. These two scalp acupoints have been used to treat a range of neurological disorders in TCM, which have a nerve conduction effect and improve cognitive impairment. Acupoint GV24 is often used in the treatment of Alzheimer's disease (Wang et al., 2020; Zheng et al., 2021), schizophrenia (Shen et al., 2014), major depressive disorder (Wu et al., 2012), and insomnia (Li et al., 2012). Moreover, the efficacy of analgesia of acupoint GV24 is internationally recognized (Cohen et al., 2017; Zhao et al., 2017, 2019). Acupoint ST8 is commonly used in treating migraine (Cayir et al., 2014; Yang et al., 2012; Zhang et al., 2014) and burning mouth syndrome (Jurisic Kvesic et al., 2015). Second, these two acupoints are easily located and accessed while the participant is lying on the scanner bed. In a routine head magnetic resonance coil, the locations of these two acupoints are covered by the coil and cannot be penetrated by acupuncturists. Our earlier invention of 12‐channel flexible head coils (HFC12) solved this problem by preserving the space to insert the needle at the scalp acupoints and connect to EAS, which is compatible with the high magnetic environment and results in real‐time stimulation during the EAS procedure (Gong et al., 2020; Tan et al., 2017; Zhan et al., 2018). Furthermore, the image quality of HFC12 is better than that of commercial 16‐ and 24‐channel rigid head coils (Kang et al., 2021). This coil has been used in clinical research (Kang et al., 2020; Liu et al., 2021a, 2021b; Luo et al., 2020; Shi et al.,2021b ; Wei et al., 2021a, 2021b).

Now, the immediate effects of EAS on scalp acupoints were not clear. We hypothesized that EAS at scalp acupoints would immediately change the brain functional connectivity. Therefore, we designed a randomized controlled study to explore the immediate effect of EAS at the scalp acupoints Shenting (GV24) and Touwei (ST8) by BOLD fMRI and independent component analysis (ICA) method.

## 2 MATERIALS AND METHODS

### 2.1 Subjects

This trial was approved by the institutional review board of Shuguang Hospital, affiliated with Shanghai University of Traditional Chinese Medicine (NO. 2017‐570‐53‐01) in accordance with the Declaration of Helsinki. This trial was registered in the Chinese Clinical Trial Registry (ChiCTR1800015692). All participants signed informed consent.

Sixty healthy individuals were recruited through advertisements for this trial at a male:female ratio of 1:1. The inclusion criteria for participants were as follows: (1) right‐handed; (2) age from 20 to 40 years; (3) healthy history; (4) no medication or acupuncture treatment one month prior to this trial; and (5) understanding of this trial and signing the informed consent form.

The exclusion criteria for participants were as follows: (1) left‐handed; (2) a history of chronic diseases, such as hypertension, diabetes, and migraine; (3) medication or acupuncture treatment one month prior to this trial; (4) fear of accepting acupuncture intervention; (5) contradictions for MR examination; (6) pregnancy; and (7) a history of head tumors or head trauma.

### 2.2 Security test

To test the influence of the electrical current of the stimulator on the magnetic field and the subjects in the MR scanner, a shield test and temperature test were performed. See Supporting Information 1 and 2 for the methods of the shield test and temperature test, respectively.

### 2.3 Randomization and blinding study design

**FIGURE 1:**
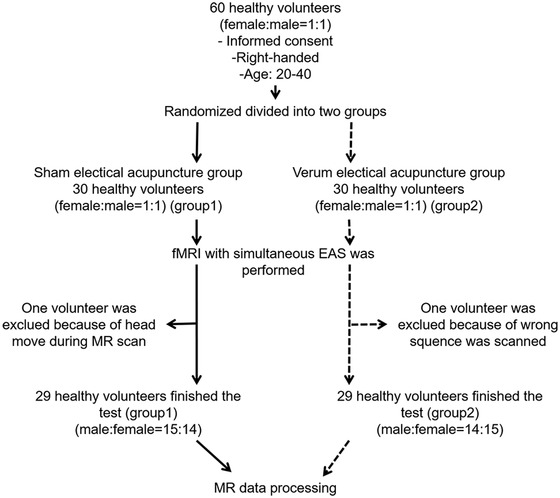
Experimental paradigm. All participants were randomly divided into two groups and then underwent fMRI with simultaneous EAS scans

First, a total of 60 subjects (female: male = 1:1) were recruited, and were divided into two groups according to gender. Each group was randomly numbered 1–30, the two groups numbered 1–15 were combined into the sham electrical acupuncture group (Group 1), and the two groups numbered 16–30 were combined into the verum electrical acupuncture group (Group 2) (Figure 1), The participants in Group 1 and Group 2 were blinded. Acupuncturists could not be blinded to the EAS assignments given the nature of the interventions. All participants underwent fMRI with simultaneous sham or verum EAS. After the scan, the extent of sourness, numbness, fullness, heaviness, spread, dull pain, sharp pain, and other feelings of every subject by visual analogue scale (VAS) (Price et al., 1983) was recorded as rated on 10‐grade scales (0 indicates that the particular feeling was not experienced, and 10 indicates that the feeling was intolerable).

### 2.4 Flexible head coil and acupuncture needles

**FIGURE 2:**
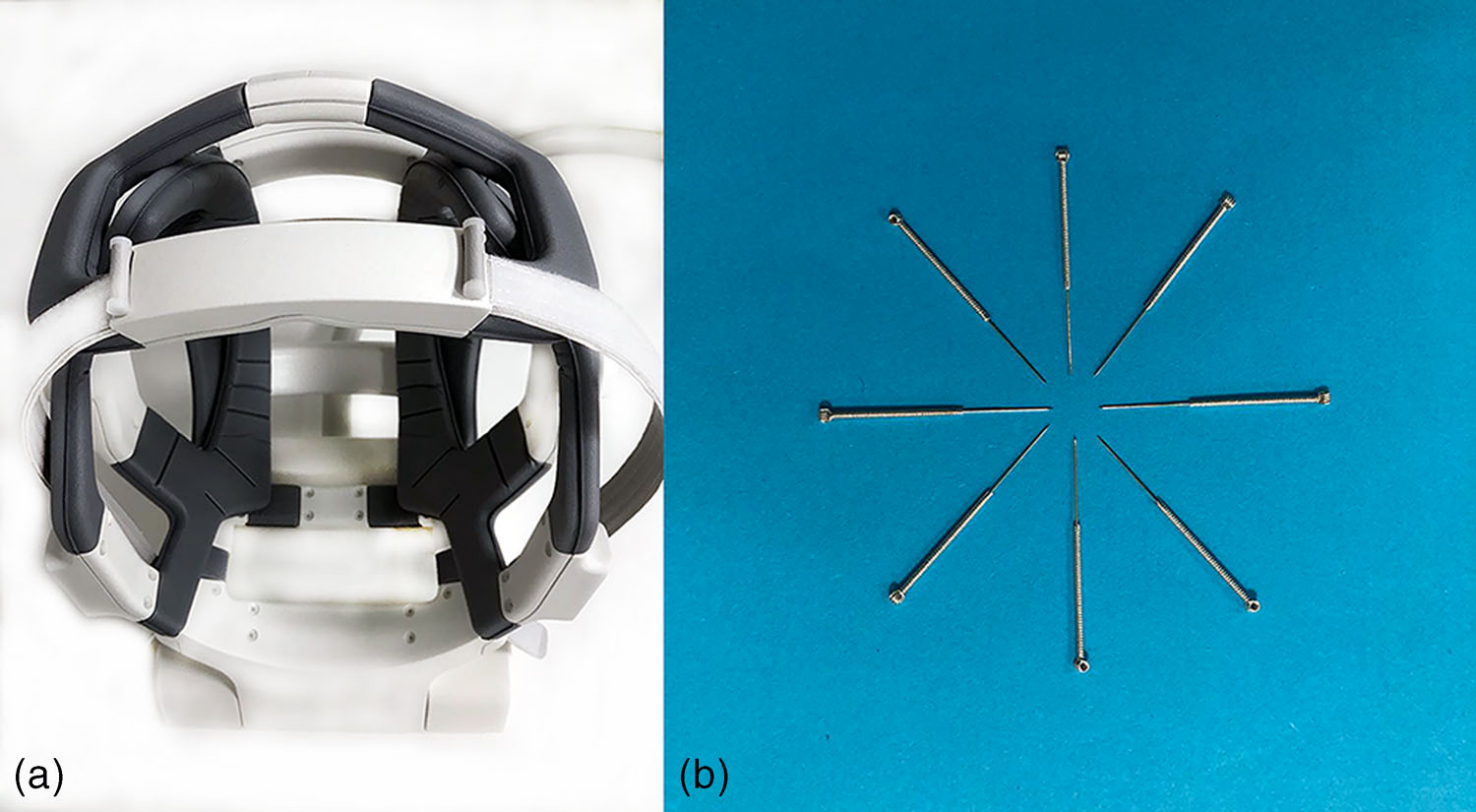
Coil and acupuncture needles were used in this study. (a) HFC12: 12‐channel array flexible head coil; (b) Gold needles: the needles were sterile, 14K gold and nonmagnetic

A 12‐channel array flexible head coil was used, with which scalp acupuncture stimulation can be simultaneously performed during the MR scanning (Figure 2a). Gold acupuncture needles with a diameter of 0.25 mm and a length of 13 mm (Huatuo Brand, Suzhou Medical Inc, China) were used for acupuncture (Figure 2b). All the operations of inserting needles and EAS were performed by the same acupuncture specialist (YL), who had 14 years of experience in acupuncture.

### 2.5 Acupoints and EAS paradigm

**FIGURE 3:**
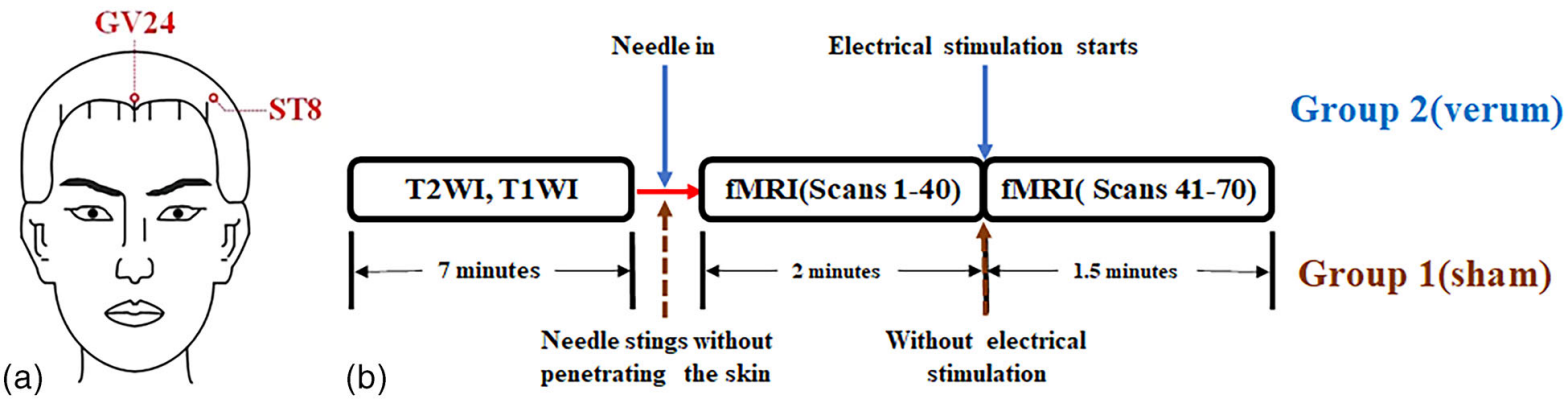
(a) The location of GV24 and ST8 acupoints. (b) Paradigm of MRI scan: The whole procedure included three steps: T1WI and T2WI (before needle intervention), 1–40 scans of BOLD fMRI, and 41–70 scans of BOLD fMRI

EAS was performed at acupoints Shenting (GV24) and Touwei (ST8), and the EAS paradigm used in this study is listed in Figure 3. According to “Nomenclature and location of acupuncture points” (GB/T 12346‐2006), acupoint GV24 was located straight up 0.5 inches in the middle of the anterior hairline. Acupoint ST8 (Left) was located on the left side of the head, 0.5 inches above the anterior hairline, and 4.5 inches left to the midline of the head (Figure 3a).

The EAS paradigm used in this study is as follows: The day before the scan, the electrical current test was performed on the participants of group 2, and the intensity of the current which depended on the endurance of the participants was recorded. All participants were asked to stay awake and try not to think of anything during brain fMRI scanning. The participants lay on the scanner bed. First, subjects in both Group 1 and Group 2 completed T1‐weighted imaging (T1WI) and T2‐weighted imaging (T2WI) scans, which took 7 min in total. Then, the scans were suspended, and the scanner bed was subsequently moved out of the scanner. Verum and sham acupunctures were performed on Group 2 and Group 1, respectively. In Group 2, gold needles were penetrated in acupoints GV24 and ST8, and the gold needle tails were connected to an EAS equipment (GV24 with positive pole and ST8 with negative pole). The current stimulation was set to start automatically after 40 scans (2 min) of BOLD scanning with the intensity according to the test results from previous day, and the electrical stimulation lasted 30 scans (1 min and 30 s). When the experiment finished, the acupuncturist recorded the acupuncture sensation. In Group 1, the dull tail of the gold needle was used to sting the same acupoints without penetrating the skin. Then, the needles were fixed in the scalp, and the tails of the needles were connected to an EAS equipment without electrical current during 70 scans (3 min and 30 s) of BOLD fMRI. This sham needle has been validated in some studies, which demonstrated that subjects could not distinguish between verum and sham needling (Kong et al., 2005; Streitberger & Kleinhenz, 1998) (Figure 3b).

### 2.6 MRI scan parameters

All participants were scanned in the supine position on a 1.5T uMR560 MR scanner (United Imaging Healthcare, Shanghai, China) with a 12‐channel head coil specially designed for scalp acupuncture (AHC12). The sequences included axial T2WI, three‐dimensional T1‐weighted imaging (3D T1WI), and blood oxygen level‐dependent fMRI. The parameters were as follows: (1) T2WI: repetition time (TR)/echo time (TE) = 5000 ms/81.1 ms, flip angle = 150°, field of view = 200 × 230 mm, slice thickness/slice gap = 5 mm/1 mm, matrix = 331 × 448; (2) 3D T1WI; TR/TE = 10.4 ms/4.4 ms, flip angle = 15°, field of view = 256 × 220 mm, thickness = 1 mm, matrix = 192 × 256, and slices = 172; (3) fMRI in axial planes: TR/TE = 3000 ms/30 ms, flip angle = 90°, field of view = 225 mm × 225 mm, thickness = 3.5 mm, slice gap = 0 mm, matrix = 64 × 64, and slices = 37, scan time = 3 min and 30 s.

### 2.7 MRI data processing

Processing of the fMRI data was performed using SPM12 (http://www.fil.ion.ucl.ac.uk/spm). For each participant, the first 10 scans were discarded from the fMRI data to avoid initial signal instability. The remaining 60 volumes were corrected for slice timing and head motion (a least squares approach and a six‐parameter spatial transformation). Subjects with (1) head motion > 2 mm in three dimensions or (2) angular rotations > 2° in three dimensions were excluded from further analysis. Subsequently, all the resultant functional images were coregistered to the individual 3D T1WI and spatially normalized to the Montreal Neurological Institute (MNI‐152) template (resampled with a voxel size of 3 × 3 × 3 mm^3^) using a unified segmentation algorithm (Ashburner, 2007). Then, the normalized images were spatially smoothed using an isotropic Gaussian filter with a full‐width‐half‐maximum (FWHM) of 6 mm to reduce spatial noise. ICA was used to further analyze these fMRI data (GIFT toolbox, http://trendscentre.org/software/gift/) (Nair et al., 2019; Porcaro et al., 2020).

### 2.8 Statistical analysis

The clinical information of the subjects was evaluated using Statistical Software and Service Solutions 23.0 (SPSS 23.0). Sex differences between the two groups were assessed using the χ2 test. Age differences between the two groups were assessed using the two‐sample *t*‐test. The Mann–Whitney U test was performed to compare the intensity of the currents and feelings associated with the needling. A statistical significance level of *p* < 0.05 was adopted.

The smoothed fMRI data were arranged into ICA of the GIFT software (http://icatb.sourceforge.net/). Using the Infomax ICA algorithm, the number of independent components (ICs) was decomposed into 20 using the MDL criteria (Li et al., 2006). The mean ICs of all the subjects and ICs for each subject were obtained from ICA separation and back‐reconstruction (Calhoun et al., 2001). The network identification was based on their spatial similarity to functional networks described in earlier studies (Di & Biswal, 2015). Six ICs were confirmed as representing known resting state networks and used as networks of interest for further analyses. Subsequently, we calculated one‐sample *t‐*test maps for all 58 subjects and all six networks. These *t* maps were then thresholded at *p* < .05 (false discovery rate [FDR]) and used as binary masks in further analyses.

To further investigate the effects produced by EAS from acupoints GV24 and ST8 and eliminate the effects of placebo, we performed a full factorial test. This result was calculated by comparing the values of FC changes (Group 1 [after sham electrical current stimulation − before sham electrical current stimulation] − Group 2 [after verum electrical current stimulation ‐ before verum electrical current stimulation]). The *p* value was set < .001 and without multiple comparison correction, and clusters with a minimum size of 20. We identified significantly different FC in the brain by overlaying the results onto the masks.

## 3 RESULTS

### 3.1 Security test

Shield test results indicated that the new electroacupuncture apparatus was compatible with the magnetic field environment, and the two devices did not affect each other, and the results of temperature test showed no extra increases in temperature near the acupuncture needles. Therefore, using an electroacupuncture apparatus in a magnetic field environment was safe. See more details in Supporting Information.

### 3.2 Clinical data

Sixty subjects were included in this study (30 subjects each in Group 1 and Group 2). In Group 1, one male subject was excluded based on movement over 2 mm. In Group 2, one female subject was excluded based on an incorrect scan sequence and refusal to repeat the scan. Sex and age in the two groups were not significantly different. The current intensity of EAS in Group 2 ranged from 1.00 to 2.00 mA. Group 2 showed more obvious feelings of sourness (*p* = .03), numbness (*p* = .02), fullness (*p* = .02), spread (*p* < .001), and sharp pain (*p* = .001). There were no significant differences in feelings of heaviness (*p* = .19), dull pain (*p* = .10), or other feelings (*p* = .32). One of the subjects in Group 2 felt euphoria with a rating of 3 (Table 1).

**TABLE 1:** Information on participants and their feelings after acupuncture

	Group 1	Group 2	Chi‐squared/T/Mann–Whitney U value	*p*
Gender (Male/Female)	15/14	14/15	0.07	.79
Age (years)^a^	25.97 ± 2.71	26.07 ± 2.03	−0.17	.87
Intensity of current (mA)^b^	0.00 (0.00–0.00)	1.00 (1.00–2.00)	24.50	< .001
Sourness^b^	0.00 (0.00–0.50)	0.00 (0.00–4.00)	298.50	.03*
Numbness^b^	0.00 (0.00–2.00)	2.00 (0.00–5.50)	280.00	.02*
Fullness^b^	0.00 (0.00–2.50)	3.00 (0.00–5.00)	280.00	.02*
Heaviness^b^	0.00 (0.00–1.00)	2.00 (0.00–3.00)	346.00	.19
Spread^b^	0.00 (0.00–0.50)	3.00 (0.50–5.00)	190.00	< .001*
Dull pain^b^	0.00 (0.00–0.50)	0.00 (0.00–3.00)	330.00	.10
Sharp pain^b^	0.00 (0.00–0.00)	2.00 (0.00–4.00)	231.50	.001*
Other feelings^b^	0.00 (0.00–0.00)	0.00 (0.00–0.00)	406.00	.32

^a^ The data are shown as the mean ± standard deviation.

^b^ The data are shown as the median (quartile).

* indicates statistical significance. Statistical level: *p*<.05.

### 3.3 fMRI with simultaneous EAS

**FIGURE 4:**
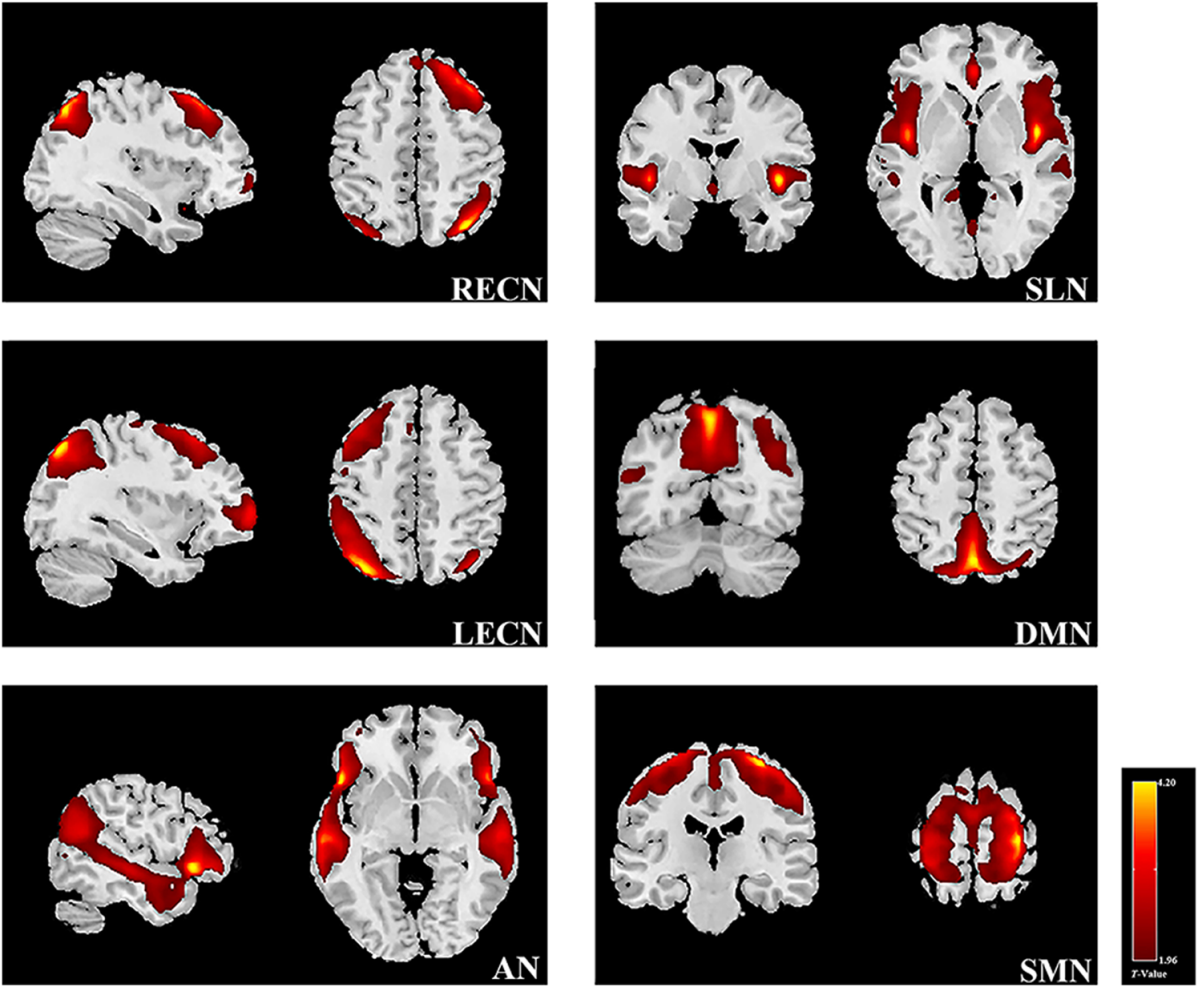
Resting‐state networks used in the time point separation analysis, which were identified by spatial ICA. The IC maps were spatially *z* transformed. RECN, right executive control network; SLN, salience network; LECN, left executive control network; DMN, default mode network; AN, attention network; SMN, sensorimotor network

In ICA analysis, six networks included the right and left executive control networks (RECN and LECN) (Glass et al., 2011), salience network (SLN) (Borsook et al., 2013), default mode network (DMN) (Baliki et al., 2008; Raichle, 2015), sensorimotor network (SMN), and attention network (AN) (Beckmann et al., 2005; Corbetta & Shulman, 2002; Farmer et al., 2012; Hemington et al., 2016; Yeo et al., 2011) (Figure 4).

**FIGURE 5:**
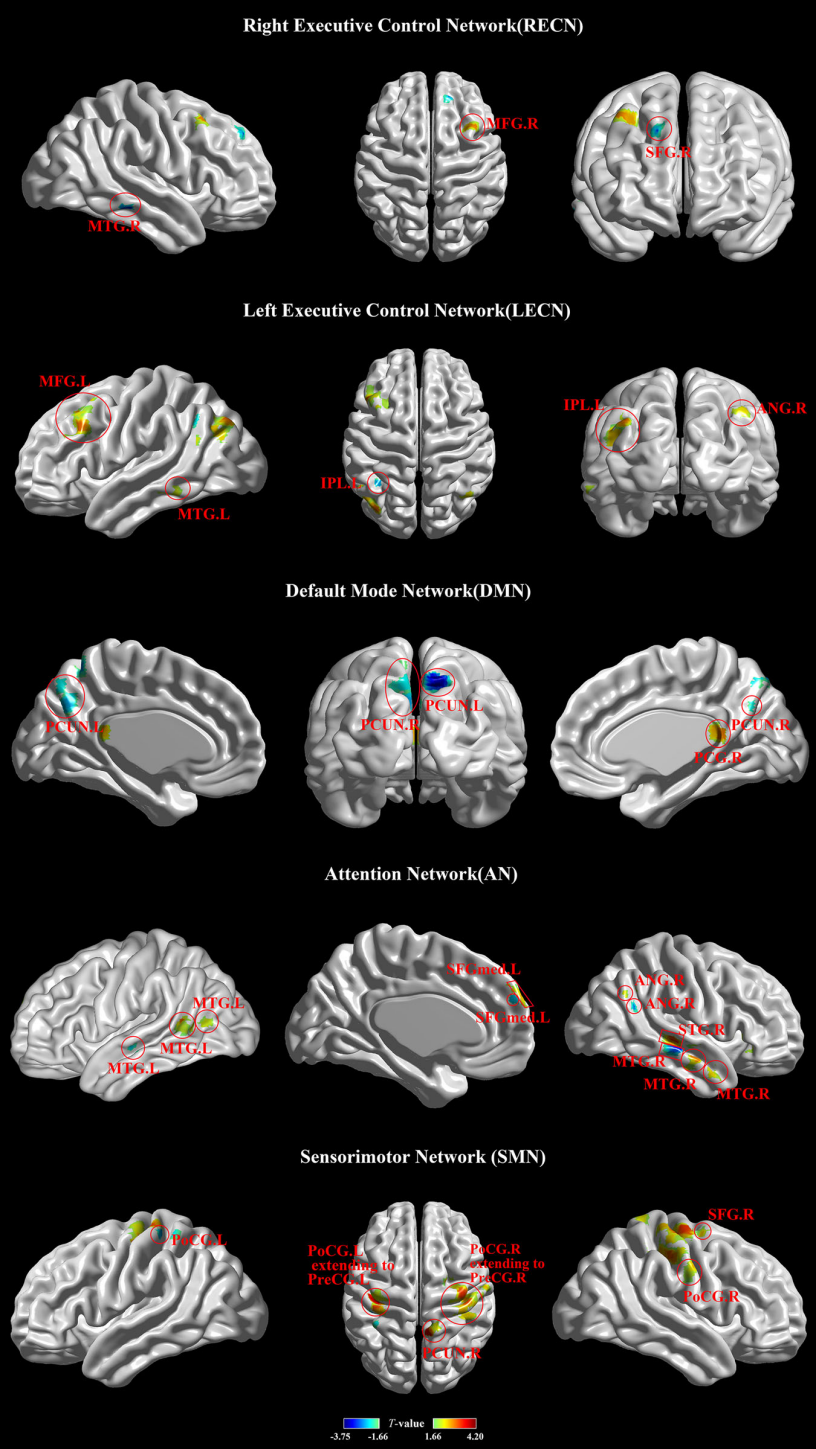
Differences in functional connectivity (FC) in the RECN, LECN, DMN, AN, and SMN (a–e) (*p* < .001, extent threshold *k* = 20 voxels). The colored brain regions with red–yellow–green indicate increased FC in the verum group with electrical stimulation, while the colored brain regions with green–blue indicate decreased FC. MTG.R, right middle temporal gyrus; MFG.R, right frontal middle gyrus; SFG.R, right superior frontal gyrus; MFG.L, left middle frontal gyrus; MTG.L, left middle temporal gyrus; IPL.L, left inferior parietal gyrus; ANG.R, right angular gyrus; PCUN.L, left precuneus lobule; PCUN.R, right precuneus lobule; PCG.R, right posterior cingulate cortex; STG.R, right superior temporal gyrus; SFGmed.L, left superior medial frontal gyrus; PoCG.R, right postcentral gyrus; PoCG.R extending to PreCG.R, right postcentral gyrus extending to right postcentral; PoCG.L extending to PreCG.L, left postcentral gyrus extending to left precentral gyrus; PoCG.L, left postcentral gyrus

In full factorial analysis, through the stimulation of placebo needles in Group 1, and excluding the stimulation of inserting needles in Group 2, the interaction results between Group 2 with electrical stimulation and Group 1 without electrical stimulation was the following: The brain FC of the right frontal middle gyrus (MFG.R) within the RECN was increased and that in the right middle temporal gyrus (MTG.R) and right superior frontal gyrus (SFG.R) was decreased in Group 2 with electrical stimulation compared to Group 1 without electrical stimulation (Table 2; Figure 5a). The brain FC in the LECN was mainly increased in Group 2 with electrical stimulation, especially in the left middle temporal gyrus (MTG.L), left inferior parietal gyrus (IPL.L), left middle frontal gyrus (MFG.L), and right angular gyrus (ANG.R), while decreased FC was observed in the IPL.L (Table 2; Figure 5b). The left precuneus lobule (PCUN.L) and right precuneus lobule (PCUN.R) within the DMN showed significant reductions in brain FC in Group 2 with electrical stimulation, while increased FC was mostly observed in the right posterior cingulate cortex (PCG.R) (Table 2; Figure 5c). In the AN, FC increases simultaneously found in the left superior medial frontal gyrus (SFGmed.L), ANG.R, MTG.L, MTG.R, and right superior temporal gyrus (STG.R) and decreased FC in the left superior medial frontal gyrus(MFGsup.L), ANG.R, MTG.L, and MTG.R (Table 2; Figure 5d). Group 2 with electrical stimulation showed significantly increased FC in the SMN, which included the left postcentral gyrus extending to the left precentral gyrus (PoCG.L extending to PreCG.L), right postcentral gyrus extending to the right postcentral gyrus (PoCG.R extending to PreCG.R), right postcentral gyrus (PoCG.R), SFG.R, and PCUN.R, and showed decreased FC in the left postcentral gyrus (PoCG.L) (Table 2; Figure 5e). There were no statistically significant FC changes in the SLN in Group 2 with electrical stimulation.

**TABLE 2:** The interactive results of the full factorial fMRI test between the sham and verum acupuncture groups

Network	AAL region	Cluster size(voxels)	MNI coordinates			BA	*t*‐Value
			*X*	*Y*	*Z*		
RECN	MTG.R	22	62	−32	−10	21	3.32
	SFG.R	58	18	48	42	9	3.06
	MFG.R	38	38	18	46	8	−2.88
LECN	MTG.L	21	−62	−32	−6	21	−2.18
	IPL.L	126	−38	−78	42	19	−3.03
	IPL.L	29	−40	−54	40	40	2.99
	ANG.R	24	42	−66	46	7	−2.35
	MFG.L	115	−44	24	34	44	−2.98
DMN	PCG.R	50	4	−46	14	29	−2.88
	PCUN.R	34	18	−60	22	31	3.06
	PCUN.L	41	−4	−66	36	7	2.48
	PCUN.R	82	12	−70	48	7	3.75
	PCUN.L	21	−8	−70	46	7	2.20
AN	MTG.R	33	54	−2	−22	21	−3.18
	MTG.R	73	64	−8	−10	21	−2.84
	STG.R	49	54	−24	−2	21	−3.20
	MTG.L	46	−50	−50	12	21	−2.98
	MTG.L	25	−58	−16	−6	22	2.30
	MTG.R	36	66	−22	−4	21	3.10
	MTG.L	23	−54	−68	14	37	−3.06
	ANG.R	22	52	−50	26	22	2.71
	SFGmed.L	21	0	54	26	10	3.07
	SFGmed.L	52	−2	64	26	10	−2.86
	ANG.R	21	58	−56	34	39	−2.76
SMN	PoCG.R	32	60	−6	40	3	−2.57
	PoCG.R extending to PreCG.R	358	38	−14	62	6	−3.27
	PoCG.L extending to PreCG.L	175	−40	−22	44	3	−4.20
	SFG.R	35	28	−6	60	6	−2.74
	PoCG.L	21	−38	−34	60	3	2.55
	PCUN.R	28	8	−48	66	5	−3.63

Abbreviations: L, left; R, right.

## 4 DISCUSSION

In this study, we first implemented fMRI with simultaneous EAS at scalp acupoints GV24 and ST8 using a specially designed HFC12 and electroacupuncture stimulator compatible with an MR scanner. The immediate changes in brain FC during stimulation at acupoints GV24 and ST8 were elucidated through these devices. Prior safety tests showed that the device we used did not interfere with magnetic field uniformity, and the increased temperature near the acupuncture point was not significant. The specific effects of EAS at acupoints GV24 and ST8 were verified by assessing the differences between the after‐stimulation sessions and pre‐stimulation sessions within the EAS group and sham acupuncture group. Therefore, the brain response areas discussed here were presumed to be “EAS‐specific effects only,” which removed the psychological components of needling. Our findings showed that EAS at acupoints GV24 and ST8 produced widespread changes in the right and left ECNs, DMN, AN, and SMN. The main effects were increased FC of the LECN and SMN and decreased FC of the DMN.

### 4.1 Emotional–cognitive networks were coordinated with each other

In general, the coordination between distinct resting‐state networks is important for efficient cognitive function, and a major challenge is to understand how these networks dynamically interact. Studies indicated (Kelly et al., 2008; Sridharan et al., 2008) that during demanding tasks where attention is directed to external information, connectivity is increased in the AN and ECN and decreased within the DMN in a tightly coupled way, which was consistent with our research results. Our research results showed that after the EAS intervention, most of the functional connectivity in the ECN and the AN were increased, while the DMN showed the opposite result. This produces an anticorrelated pattern over time between the DMN and AN/ECN. Increases in the magnitude of this anticorrelation have been associated with improved behavioral responses (Christoff et al., 2009; Humphreys & Sui, 2016). Similarly, study from Fang et al. (2012) indicated that EAS strongly decreased the local FC in the regions that belonged to the DMN and the limbic–paralimbic‐neocortical network. Executive and default networks have been suggested to act in opposition to each other, and the same relationship is thought to exist between the AN and the DMN.

Compared with some transcranial direct current stimulation (tDCS) studies, for example, our findings are directly consistent with Shahbabaie et al. (2018). They reported that single‐session bilateral tDCS alters functional connectivity of the large‐scale networks, including decreased functional connectivity in DMN and increased functional connectivity in ECN. Furthermore, more similar results from Peña‐Gómez et al. (2012) indicated active tDCS resulted in increased/reduced synchronous activity of the AN/DMN. However, except for these consistent results, heterogeneous changes within these networks were observed in our study.

These results may suggest that emotional–cognitive networks play a coordinated and balanced role in the top‐down control system. According to this model, we thus suggested that the sources of attention be switched to external stimuli but not self‐referential monitoring during EAS. That being said, the present work could also explain why the efficient results of EAS were reported in a wide range of cognitive functions.

In addition, our study also found that the same brain regions have both increased and decreased FC, which we do not think is a bias in the results. Especially, the IPL and the MTG are not only part of the ECN, but also an important part of the DMN (Raichle, 2015). This result may further illustrate the competition relationship between the two networks (DMN and ECN), and the opposite activities shown in the same brain area may prompt different subregions of the same brain area to process different network information; this hypothesis needs to be confirmed by further research.

### 4.2 The PCG might play an independent role in the DMN

Interestingly, our work showed that the FC of the PCG was increased compared to that of the other components of the DMN. This result is consistent with Keeser et al. (Keeser et al. et al., 2011). They showed increased DMN connectivity after prefrontal tDCS. The PCG represents a central node within the DMN (Smith et al., 2018). However, other evidence has suggested that this region is highly heterogeneous and may play a direct role in regulating the focus of attention (Leech & Sharp, 2014). Anatomical studies have shown that this region is not homogeneous, and electrophysiological recordings in nonhuman primates also suggest that it is directly involved in some forms of attention (Hayden et al., 2009). Leech et al. (Leech et al., 2011) found that as the degree of externally directed task difficulty increased, the dorsal PCG showed increased integration within the DMN and more anticorrelation with the cognitive control network activated by the task. This result was in accordance with the findings of Crittenden et al. (Crittenden et al., 2015). These results are consistent with our result that the PCG showed a different connectivity pattern from the other components of the DMN. Abnormalities in the PCG have been shown in a wide range of neurological and psychiatric disorders (Buckner et al., 2008; Zhang & Raichle, 2010). Task functional neuroimaging and positron emission tomography studies have shown task‐dependent deactivation of the PCG and reduced metabolism in a range of neurological and psychiatric disorders, including Alzheimer's disease (Sun et al., 2012), schizophrenia (Wu et al., 2014), and depression (Ho et al., 2015). Therefore, the increased connectivity of the PCG in this study may be related to the effects of EAS on neuropathological mechanisms in the treatment of the above diseases. In addition to the DMN, the PCG has been shown to be involved in the dorsal AN and the frontoparietal control network.

### 4.3 A network‐specific enhancement of connectivity following motor cortex EAS

We found that increased FC was mainly concentrated in the SMN. The SMN plays a vital role in organization and preparation before spontaneous exercise. It was confirmed that changes in electroencephalography (EEG) in the SMN occurred 1−2 s earlier than the start of exercise (Nguyen et al., 2014). The postcentral gyrus is the main sensory area of the brain (Kalenine et al., 2010). It is activated during tasks related to motion recognition and execution. Therefore, it has been regarded as one of the key brain regions for processing motor visual information. The present study also found that EAS increased connectivity of the bilateral postcentral gyrus, which might allow the body to prepare for sports activity and help recover motor function, especially in patients after stroke (Chae et al., 2013; Dhond et al., 2008).

### 4.4 fMRI with simultaneous EAS and eliminating placebo effects could reflect the true brain effects of EAS

It has been speculated that altered function of brain networks might show special modulatory effects of EAS, which might offer a possible explanation for EAS‐related effects in central nervous system diseases. Although previous studies have indicated that EAS specifically regulates the cerebral functional network, the exact differences between verum and sham EAS are not clearly understood. First, in recent years, more than half of the research teams have chosen the multiple block design method to perform fMRI related to acupuncture (Petersen & Dubis, 2012). Specifically, multiple acupuncture operations have been placed in an experimental module to collect BOLD signals in the baseline and acupuncture states. However, some researchers (Li et al., 2016) believe that due to the continuous effect of acupuncture, the baseline level of brain function connectivity of the subject gradually increases during the continuous acupuncture process, and the increase in the baseline level in the block design introduces bias into the research results. To solve these problems, we performed only one block of rest and then one block of sham/verum stimulation in this study to investigate the effects produced only by EAS for acupoints and eliminate the effects of inserting the needles. Second, the effects of the EAS ritual consist of psychological components (e.g., needling credibility based on anticipation, imagination, or concentration) and the electrical current stimulation of acupoints. Many controlled studies have shown that stimulation with real acupuncture and Steitberger needles evokes heterogeneous modulation patterns in whole functional brain networks (Lee et al., 2019; Makary et al., 2018; Sun et al., 2014). The “specific” brain network reactions evoked by EAS have remained unclear. This trial allowed us to experimentally dissociate acupuncture needling‐specific and nonspecific components. Specifically, real acupuncture was designed to include the psychological components and “specific” effects, while sham acupuncture was designed as a control that involved only the placebo effects. This study investigated the associations between stimulation by EAS alone (controlling for the influence of psychological components) and brain functional connectivity. Based on the present study, EAS was especially related to activating higher‐order perception and fine regulation of sensorimotor function.

### 4.5 fMRI with simultaneous EAS may become a new method for searching the target scalp acupuncture for treatment of neurological and psychiatric diseases

Scalp acupuncture is a modality of acupuncture in which acupuncture needles are inserted into a certain depth of the scalp in order to affect the function of corresponding areas of the cerebral cortex and relieve symptoms. Different brain regions are responsible for corresponding functions. For example, MTG is concerned with cognitive processes such as language and semantic memory processing, and multimodal sensory integration (Mesulam, 1998). IPL is mainly involved in the response to pain and the sensation of temperature and pressure (Seghier, 2013). Identifying key cortical areas associated with the immediate stimulation on a certain acupoint may shed light on applying scalp acupuncture for the treatment of diseases. In this study, GV24 and ST8 are located near the frontal and temporal lobes cortex; the cortex changes are also consistent with the results of several studies on GV24 or ST8 acupoints in the treatment of cognitive and executive dysfunction (Yang, 2021), memory loss (Lin, 2020), and pain‐related diseases (Yang et al., 2012). Another study integrated meta‐analysis, resting‐state functional connectivity, and diffusion tensor imaging, and found that the prefrontal cortex, the middle temporal gyrus, the temporal pole, the supplementary motor area, the inferior occipital gyrus, and the precuneus were involved in the pathophysiology of dementia (Cao et al., 2020). There is a large overlap with the changes in brain regions that we found with acupuncture at GV24 and ST8, so these two points should be considered in the scalp acupuncture programs for the treatment of dementia. Consistently, GV24 is often used in prescriptions as a common acupuncture point (Cao et al., 2020). Therefore, the real‐time changes in brain regions induced by acupuncture may help guide clinical acupoint selection.

### 4.6 Limitations

Our study has some limitations. First, the subjects in the current research were healthy people, but the application of EAS is aimed at patients with different diseases, and the current study is a small sample size study. Therefore, in the future, studies with a large sample size should be conducted with target groups of patients with certain diseases (depressive disorder, migraine, etc.). Second, for the group with real EAS, the needles were inserted into the skin and adjusted until the subjects experienced the deqi sensation. This process itself usually induces some degree of immediate pain due to the operation of twisting needles and the body's biochemical response to tissue damage. For sham EAS, in addition to producing the same psychological placebo effect, sham acupuncture needles produced a certain somatosensory touch that produced a stinging sensation at the same acupoints without penetrating through the skin. These two types of tactile stimulation might also produce a little different brain response, and Group 1 adopts non‐current stimulation, which increases the limitations of this study. In future, it is necessary to study the effect of current stimulation on the non‐acupoint group. Third, the scan time for the fMRI was 3 min and 30 s, and the MRI system was 1.5 T. These factors reduced the power to find changes in brain FC after EAS, so the signal‐to‐noise ratio is relatively low. To get more positive results, a lenient *p*‐value was set in our analysis, increases likely of false‐positive results (Eklund et al., 2016), and because we focused on the supratentorial network, the changes of cerebellum were not included in this study. Finally, in this study, the control group was a thorough non‐stimulation sham acupuncture, and control research on non‐meridian and non‐acupoints requires further experiments in the future. Longer scan times and higher magnetic field should be used in future studies to confirm the results of this study.

## 5 CONCLUSION

Our research provides a new approach to study the real‐time effects of EAS at scalp acupoints on the brain. The results suggest that EAS at scalp acupoints mainly induced increased FC of brain networks involved in cognitive function. The next important step in EAS research is to explore the neural mechanisms of EAS when used in the context of diseases or symptoms for which it has been claimed that EAS therapy is effective. In future, we can also study specific acupuncture and moxibustion stimuli that cause corresponding patterns of brain increased or decreased FC to guide the treatment of patients with central nervous system diseases.

## AUTHOR CONTRIBUTIONS

All the listed authors have participated actively in the study. Wenli Tan, Kay Jann, and Yijing Li designed the study; Hui Chen, Jiling Huang, Yilei Chen, Yingjie Kang, Zhigang Gong, Yanwen Huang, and Hui Wang collected the data; Wenli Tan, Hui Chen, and Kay Jann analyzed the data; Hui Chen and Wenli Tan wrote the manuscript; Kay Jann revised the manuscript. Songhua Zhan provided technical support.

## CONFLICT OF INTEREST

The authors declare that the research was conducted in the absence of any commercial or financial relationships that could be construed as a potential conflict of interest.

### PEER REVIEW

The peer review history for this article is available at https://publons.com/publon/10.1002/brb3.2829.

## Supporting information

Fig S1. The waveform produced by the electroacupuncture apparatus. The waveform was normal.
Fig S2. The impact of the electroacupuncture apparatus on the uniformity of the B0 and B1 fields in the transverse, sagittal and coronal orientations.
Fig S3. The impact of the electroacupuncture apparatus on the vortex in the transverse, sagittal and coronal orientations.
Table S1. Results of the test assessing increases in temperature during the MR scan
**Table S2**. The results of the correlations between network measurements and feelings that were significantly different after acupuncture within the two groups

## Data Availability

The original data which support the conclusions of our study could be gotten from the corresponding authors with sound reasons.
