# Supplementary material for: A true response of the brain network during electroacupuncture stimulation at scalp acupoints: An fMRI with simultaneous EAS study
Source: Brain Behav. 2022 Nov 25;13(1):e2829. doi: 10.1002/brb3.2829 (PMC9847615; doi:10.1002/brb3.2829)
Supplement: Supplementary file 1 — Fig S1. The waveform produced by the electroacupuncture apparatus. The waveform was normal. Fig S2. The impact of the electroacupuncture apparatus on the uniformity of the B0 and B1 fields in the transverse, sagittal and coronal orientations. Fig S3. The impact of the electroacupuncture apparatus on the vortex in the transverse, sagittal and coronal orientations. Table S1. Results of the test assessing increases in temperature during the MR scan Table S2. The results of the correlations between network measurements and feelings that were significantly different after acupuncture within the two groups [file BRB3-13-e2829-s001.docx]

Supplementary Material

1. ***Shield Test***

**1.1 Test design**

To test the influence of the electroacupuncture apparatus in a magnetic resonance environment, we initially slowly took the apparatus into the 1.5T magnetic resonance scanning room and turned on 220 VAC power. We found that the output pulse voltage of the electroacupuncture apparatus in the magnetic resonance environment was unstable, and the power supply was also unstable. At the same time, the output signal had an impact on the uniformity of the magnetic field. To solve this problem, we performed a series of improvements, which mainly included the following: 1. removing the internal AC-DC power conversion module of the electroacupuncture apparatus and converting it into four batteries; 2. designing an electroacupuncture apparatus shielding shell by using mechanical Solidworks software; 3. designing, simulating, and manually welding the band-block filters to address the interference signals, which were produced from the output electrical signals of the low-frequency pulse generator and affected the magnetic field; 4. maintaining a safe distance of more than one metre between the electroacupuncture apparatus and the magnetic field; and 5. transferring the output signal from the electroacupuncture apparatus through a coaxial RF cable with a trap made of RG316 material, which was easy to bend.

**1.2 Results**

The new electroacupuncture apparatus was compatible with the magnetic field environment, and the two devices did not affect each other. First, the electroacupuncture apparatus could output electrodynamic pulses in a magnetic field environment in a normal manner (Fig S1). Second, there were no effects on the uniformity in the B0 and B1 fields from the three orientations (Fig S2). Finally, no effects were found in the gradient vortex of the MR system from the three orientations (Fig S3).

**
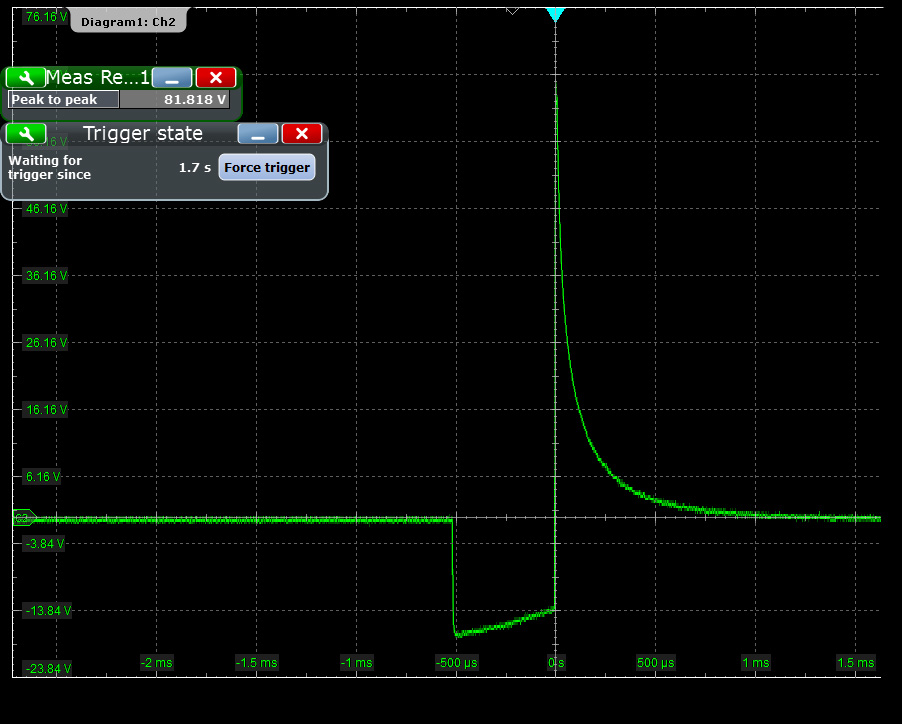
**

Fig S1. The waveform produced by the electroacupuncture apparatus. The waveform was normal.

**
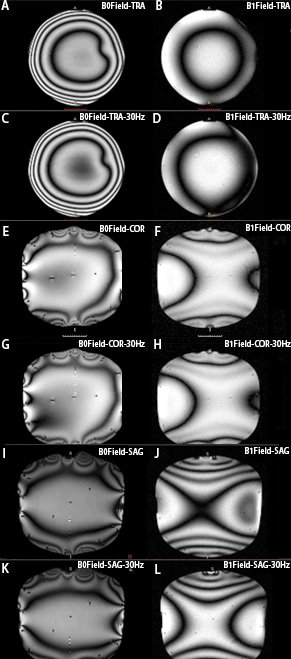
**

Fig S2. The impact of the electroacupuncture apparatus on the uniformity of the B0 and B1 fields in the transverse, sagittal and coronal orientations. A-D: there was no impact on B0 and B1 fields in the transverse section; E-H: there was no impact on the B0 and B1 fields in the coronal section; I-L: there was no impact on the B0 and B1 fields in the sagittal section.

**
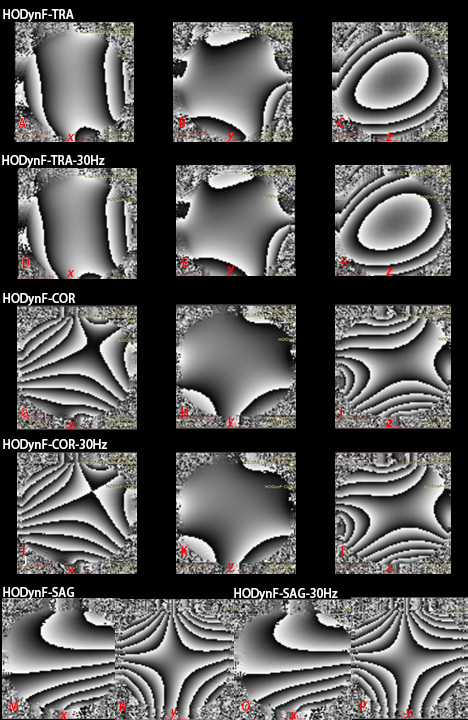
**

Fig S3. The impact of the electroacupuncture apparatus on the vortex in the transverse, sagittal and coronal orientations. A-D: there was no impact on the vortex in the transverse section; E-H: there was no impact on the vortex in the coronal section; I-L: there was no impact on the vortex in the sagittal section.

**1.3 Conclusion**

Our test indicated that the electroacupuncture apparatus was compatible with being in a magnetic resonance environment, the electroacupuncture apparatus did not significantly impact magnetic resonance imaging, and the magnetic field environment had no impact on the operation of the electroacupuncture apparatus.

***2.*** ***Temperature Test***

**2.1 Test design**

In the temperature test, 4.0 lb of pork was placed on the MR scanner bed, and two gold needles penetrated the pork in the fat area (needle 1) and muscle area (needle 2). Two optical fibre temperature probes (probe 1 and probe 2) were placed beside the acupuncture needles, and the other two probes (probe 3 and probe 4) were placed in the muscle of the pork away from the needles. There were three rounds of MR scans with the following sequences. All data were obtained from the same 1.5T uMR560 MR scanner (United Imaging Healthcare, Shanghai, China) with a 12-channel head coil specially designed for scalp acupuncture (AHC12). The sequences in each round included axial T1WI, T2WI, sagittal 3D T1WI, and three BOLD fMRI scans with a total scan time of 40 minutes. The parameters were as follows: (1) T2 scan with TR/TE = 5000/81.1 ms/ms, flip angle = 150°, field of view = 200×230 mm, slice thickness/slice gap = 5 mm/1 mm, matrix = 331x448; (2) T1 scan with TR/TE = 10.4/4.4 ms/ms, flip angle = 15°, field of view = 256×220 mm, thickness = 1 mm, matrix = 192×256, and slices = 172; (3) fMRI in axial planes with TR/TE = 3000/30 ms/ms, flip angle = 90°, field of view = 225 mm ×225 mm, thickness = 3.5 mm, slice gap = 0 mm, matrix = 64×64, and slices = 37. The fourth MR scan included only ten minutes of BOLD fMRI.

**2.2 Results**

The temperature of the MR scanner room was 22 °C. In the test assessing an increase in temperature, the temperature in the fat and muscle with the acupuncture needle rose 1.46 °C and 3.39 °C after the first round of MR scans, while it increased 1.73 °C and 1.65 °C in muscles without the acupuncture needles. In the second round of the test, the temperature rose 2.00 °C and 1.15 °C in the acupuncture needle area and 1.76 °C and 1.70 °C in the area without the acupuncture needle. In the third round of the test, the respective temperatures were -0.15 °C, 1.00 °C, 1.39 °C, and 0.12 °C. In the fourth round of the test, the temperature changes were 0.12 °C, 0.03 °C, 0.22 °C, and 0.03 °C (Table S1).

Table S1. Results of the test assessing increases in temperature during the MR scan

|  |  | Temperature Probe 1  (in fat） | Temperature Probe 2  (in muscle) | Temperature Probe 3  (in muscle) | Temperature Probe 4  (in muscle) |
| --- | --- | --- | --- | --- | --- |
| First round  (40 minutes) | T0 (℃) | 14.68 | 13.33 | 16.19 | 16.17 |
|  | T1 (℃) | 16.14 | 16.72 | 17.92 | 17.82 |
|  | ΔT (℃) | 1.46 | 3.39 | 1.73 | 1.65 |
| Second round  (40 minutes) | T0 (℃) | 17.41 | 19.05 | 18.57 | 17.6 |
|  | T1 (℃) | 19.41 | 20.20 | 20.33 | 19.3 |
|  | ΔT (℃) | 2.00 | 1.15 | 1.76 | 1.70 |
| Third round  (40 minutes) | T0 (℃) | 20.43 | 20.18 | 20.77 | 20.19 |
|  | T1 (℃) | 20.28 | 21.18 | 22.16 | 20.31 |
|  | ΔT (℃) | -0.15 | 1.00 | 1.39 | 0.12 |
| Fourth round  (10 minutes) | T0 (℃) | 20.28 | 21.00 | 22.31 | 20.31 |
|  | T1 (℃) | 20.40 | 21.03 | 22.53 | 20.34 |
|  | ΔT (℃) | 0.12 | 0.03 | 0.22 | 0.03 |

T0 indicates the original temperature before the test, T1 indicates the temperature after the test, and ΔT indicates the change in temperature during the test.

**2.3** **Conclusion**

When connecting the electroacupuncture apparatus (with traps) with acupuncture needles, the most obvious changes in temperature were found near the acupuncture needles in the first round of the test, with an increase of 3.39 °C. After another three rounds of scans, the changes in temperature near the acupuncture needles were not significantly higher than the changes in temperature in the muscles without acupuncture needles. Therefore, the temperature increases near the acupuncture needles in the first-round scan might have been due to the lower initial temperature of the pork and the absorption of heat from the environment. Therefore, using an electroacupuncture apparatus in a magnetic field environment is safe and does not show extra increases in temperature near the acupuncture needles.

1. ***Relationships between specific feelings after acupuncture and particular brain areas***

**3.1** **Correlation analysis**

To understand the relationships between the feelings induced by needling and the change of network connectivity, partial correlations were used to evaluate the correlation between the two groups with significant differences in the acupuncture feeling scores and the above network measurements with age, sex and the intensity of the current added as covariates. Here, *p*<0.05 was used as an exclusive significance level. Moreover, *p*< 0.05 with Bonferroni correction was used to correct for the number of statistical tests for the network measurements of the same nature.

- 1. **Results**

The different feelings experienced during acupuncture were related to one or several specific brain areas. Sourness and numbness scores after acupuncture were negatively correlated with ANG.R in the AN (r=-0.274, p=0.043; r=-0.314, p=0.019, respectively). Fullness scores after acupuncture were negatively correlated with the SFG.R in the RECN (r=-0.293, p=0.030) and the IPL.L in the LECN (r=-0.307, p=0.022; r=-0.279, p=0.039, respectively) and positively correlated with the PreCG R in the SMN (r=0.292, p= 0.031). Spread scores after acupuncture were positively correlated with the SFG.R in the SMN (r=0.382, p=0.004). Sharp pain scores after acupuncture were negatively correlated with the IPL.L in the LECN (r=-0.302, p=0.025), PCUN.R in the DMN (r=-0.271, p=0.045), and SFGmed.L in the AN (r=-0.272, p=0.045) (Table 3). However, correlations of all of the network parameters to the feelings after acupuncture did not survive after Bonferroni correction( Table S2).

**Table S2**. The results of the correlations between network measurements and feelings that were significantly different after acupuncture within the two groups

|  |  | Sourness | Numbness | Fullness | Spread | Sharp pain |
| --- | --- | --- | --- | --- | --- | --- |
| RECN | MTG.R | *--* | -- | -- | -- | -- |
|  | SFG.R | *--* | -- | *r*=-0.293 | -- | -- |
|  | MFG.R | *--* | *--* | -- | -- | -- |
| LECN | MTG.L | *--* | -- | -- | -- | *--* |
|  | IPL.L | -- | -- | *r*=-0.307 | -- | *r*=-0.302 |
|  | IPL.L | *--* | -- | *r*=-0.279 | -- | -- |
|  | ANG.R | -- | -- | -- | -- | -- |
|  | MFG.L | *--* | *--* | -- | -- | *--* |
| DMN | PCG.R | -- | -- | -- | -- | -- |
|  | PCUN.R | -- | -- | -- | -- | -- |
|  | PCUN.L | -- | -- | -- | -- | -- |
|  | PCUN.R | -- | -- | -- | -- | *r*=-0.271 |
|  | PCUN.L | -- | -- | -- | -- | -- |
| AN | MTG.R | -- | -- | -- | -- | -- |
|  | MTG.R | -- | -- | -- | -- | -- |
|  | STG.R |  |  |  |  |  |
|  | MTG.L |  |  |  |  |  |
|  | MTG.L | -- | -- | -- | -- | -- |
|  | MTG.R | -- | -- | -- | -- | -- |
|  | MTG.L | -- | -- | -- | -- | -- |
|  | ANG.R | -- | -- | -- | -- | -- |
|  | SFGmed.L | -- | -- | -- | -- | -- |
|  | SFGmed.L | -- | -- | -- | -- | *r*=-0.272 |
|  | ANG.R | *r*=-0.274 | *r*=-0.314 | -- | -- | -- |
| SMN | PoCG.R | -- | -- | -- | -- | -- |
|  | PoCG.R extending to PreCG.R | -- | -- | *r*=0.292 | -- | -- |
|  | PoCG.L extending to PreCG.L | -- | -- | -- | -- | -- |
|  | SFG.R | -- | -- | -- | *r*=0.382 | -- |
|  | PoCG.L | -- | -- | -- | -- | -- |
|  | PCUN.R | -- | -- | -- | -- | -- |

L: left; R: right

*Statistical threshold is *p*<0.05, -- indicates no statistical significance.

**3.3 Conclusion**

Regarding the significance of the current network findings after acupuncture to clinical outcomes, associations were found between brain activation areas and feelings after acupuncture. The present study confirmed that different feelings after acupuncture have different impacts on brain functional effects. In the past 10 years, research exploring the effects of different sensations after acupuncture on brain functional activity has gradually increased. In addition, the majority of participants were healthy people. The importance of collecting and recording needle sensation data in neuroimaging studies of acupuncture has been shown(Asghar et al., 2010; Shi et al., 2016). For example, Fang et al. found that deqi and sharp pain during acupuncture at Taichong elicited opposite patterns in functional brain network activity(Fang et al., 2012). Sharp pain indicates a harmful stimulus that results in a brain functional network that is relatively resistant and should be avoided in fMRI deqi studies(Zhang et al., 2018). Zhu et al.(Zhu et al., 2009) found that when the acupuncture acupoint Taixi was stimulated, there was often a difference between sourness and numbness. Since numbness usually causes limb movement, the fMRI test should ensure that the subject does not have any physical activity; otherwise, this movement will result in false positives and distort the results. Therefore, identification of the changes in the corresponding brain regions produced by various sensations is helpful for our ongoing clinical analyses.

**References:**

Asghar, A. U., Green, G., Lythgoe, M. F., Lewith, G., andMacPherson, H. (2010). Acupuncture needling sensation: the neural correlates of deqi using fMRI [Journal Article; Research Support, Non-U.S. Gov't]. BRAIN RESEARCH, 1315, 111-118. http://doi.org/10.1016/j.brainres.2009.12.019

Fang, J. L., Kathleen, H. K., Erika, N., Zhou, L. K., Wang, X. L., andZhu, B. (2012). Deqi and sharp pain during acpuncture at Taichong elicting the opposite functional brain network effects-an fMRI study. Chinese Journal of Integrated Traditional Chinese and Western Medicine Imaging, 10, 4-9.

Shi, Y., Zhang, S., Li, Q., Liu, Z., Guo, S., Yang, J., andWu, W. (2016). A study of the brain functional network of Deqi via acupuncturing stimulation at BL40 by rs-fMRI [Journal Article; Research Support, Non-U.S. Gov't]. COMPLEMENTARY THERAPIES IN MEDICINE, 25, 71-77. http://doi.org/10.1016/j.ctim.2016.01.004

Zhang, Z., Wang, Y. F., Sun, J. J., Hu, N. J., Wu, G. W., Hu, S. Q., Wang, P., Zhao, M. Y., Ma, L. X., andZhu, J. (2018). Progress of fMRI-based deqi research in recent 10 years. Chinese Acupuncture & Moxibustion, 38, 445-450.

Zhu, F., Cheng, H. J., Chen, S. J., andLiu, J. W. (2009). Evaluation of Needle Sensation in fMRI Test of Stimulating Taixi Point. Shenzhen Journal of Integrated Traditional Chinese and Western Medicine, 19, 107-109.
